# A Developmental Psychopathology Perspective on Political Violence and Youth Adjustment

**DOI:** 10.3390/ijerph20105864

**Published:** 2023-05-18

**Authors:** Bethany Wentz, Laura E. Miller-Graff, Christine E. Merrilees, E. Mark Cummings

**Affiliations:** 1 Department of Psychology, William J. Shaw Center for Children and Families, University of Notre Dame, Notre Dame, IN 46556, USA; 2 Kroc Institute for International Peace Studies, University of Notre Dame, Notre Dame, IN 46556-5677, USA; 3 SUNY Geneseo, Geneseo, NY 14454, USA

## 1. Introduction

According to the United Nations (2021), [1] over 1 billion children are exposed to violence worldwide each year. However, research on this vital issue, especially in the area of political violence, has typically been limited to documenting the risk that exposure to violence poses for developing adjustment problems. Thus, scientific understanding is underdeveloped, and approaches to intervention have limited scientific foundations [2]. A developmental psychopathology perspective provides a basis for more rigorously identifying the processes that underlie risk for adjustment problems and pathways that may lead to resilience, as well as well-developed avenues for translating research findings into more efficacious approaches to intervention. Accordingly, this Special Section includes innovative research that advances tests of process-oriented explanatory models, including longitudinal tests, as well as new directions for developing more efficacious interventions based on the translational intervention model espoused by the developmental psychopathology perspective.

The impact of violent ethnopolitical conflict on the well-being of youth has become an international concern. An increasing number of researchers and policymakers have turned their attention to youth affected by violent conflicts [3]. This Special Section focuses on youth because they are an understudied and vitally important population in numerous contexts affected by political violence and armed conflict. The study of youth exposed to political violence and armed conflict is pertinent to the promotion of healthy, adaptive development of youth and long-term positive outcomes for communities at large, including the prevention of reemerging violent conflict over time [4].

The substantial risks and challenges violent contexts pose to youth development are well-established, including studies that have documented the risk for negative outcomes related to exposure to political violence, including externalizing problems (such as aggression), internalizing disorders (such as depression), and posttraumatic stress symptoms [2]. However, individual outcomes in these contexts vary widely. For example, some youth exhibit resilience in the face of threats and challenges related to living in contexts of political violence and armed conflict [3,5]. Given the wide range of documented outcomes, further investigation is needed concerning the processes and factors accounting for the differential relations between political violence and youth development, including identifying the risk factors that relate to maladjustment or protective factors that foster positive outcomes [6].

In recent years, excellent reviews have been devoted to this topic [7,8,9]. This Special Section aims to contribute to the ongoing conversation by presenting research concerned with multiple outcomes and processes and multiple research designs, including new intervention approaches and trials and longitudinal research. The developmental psychopathology perspective provides a basis for researchers to uncover the processes underlying adaptive development as well as the development of psychopathology, as well as approaches to more effective prevention and intervention. This Special Section presents new research that advances the study of political violence, including the related issue of migration and child adjustment, both from the perspective of process-oriented empirical research and research that translates relevant theory and findings of research into approaches to prevention/intervention.

## 2. Theoretical Models

At the outset, theoretical models pertinent to framing the empirical articles included in the Special Section are briefly outlined below.

## 3. Developmental Psychopathology

Developmental psychopathology focuses on the identification of the dynamic processes that underlie the course of development. To that end, psychopathology is considered a deviation from typical development and thus must be considered in relation to typical development [10]. It follows that in order to understand psychopathology, one must simultaneously seek to understand the processes underlying typical development in order to capture what processes went awry and ultimately led to maladaptation. Furthermore, because disorders have long histories and predictors that emerge before the ultimate outcome of psychopathology, the identification of predictors of pathology is key from a developmental psychopathology perspective. At the same time, this framework also allows for the exploration of predictors of later adaptation [11].

## 4. Social Ecological Systems Theory

The social ecological systems theory posits that development is nested within multiple systems that contribute to an individual’s specific pathway of development [12,13]. The microsystem is the individual’s immediate environment, including home, school, neighborhood, and interpersonal interactions among individuals. The mesosystem refers to the interaction among two microsystems (e.g., the interaction between home and school). The ecosystem refers to external sources that impact an individual indirectly, such as a parent’s job, the local school board, or government agencies. The macrosystem refers to aspects such as broader cultural influences, political systems, and the larger society. Lastly, the chronosystem refers to all experiences across the lifespan, including major life transitions and historical events. By considering several contexts of the social ecology, a more holistic understanding of the effects of political violence on youth can be achieved [14,15,16,17].

## 5. Emotional Security Theory

The emotional security hypothesis put forth by Davies and Cummings (1994) posits that emotional security is a regulatory process through which children maintain their perceived sense of safety through their emotional, behavioral, and cognitive responses to threats. Emotional Security Theory (EST) was initially conceptualized as a response specifically to inter-parental conflict. However, the construct has since been extended as a psychological response to general family conflict and contexts of broader societal conflict, including ethnopolitical violence [18,19]. A surfeit of empirical work over the past few decades has documented positive relationships between emotional security about interparental, family, and community relationships and greater family cohesion, less family conflict, and fewer child adjustment problems, as well as other positive outcomes [19,20,21,22,23]. Emotional insecurity, on the other hand, has been linked with many negative outcomes [6,14,18]. Increases in family conflict and community conflict, whether sectarian or nonsectarian, undermine adolescents’ sense of security, including parent–adolescent attachment security, emotional security in the family, and an adolescent’s sense of security in the community. Ultimately, emotional insecurity in any of these contexts contributes to adolescents’ internalizing and externalizing problems [6,24]. Notably, the significance of emotional insecurity to outcomes for adolescents affected by violence has been implicated in multiple contexts of violent tension [25,26,27]. Emotional security is thus crucial for understanding socio-emotional functioning, particularly in the contexts of sectarian violence and tension [2,4].

## 6. Social Cognitive Ecological Framework

The work of Huesmann, Dubow, and colleagues is underpinned by a theoretical framework for understanding how persistent and extreme exposure to ethnopolitical conflict interacts with cognitive, emotional, and self-processes to influence youth adjustment. This framework is guided by three main theoretical threads [28,29]. The first thread focuses on how observational and social learning processes influence the development of social-cognitive processes that affect behavior, emphasizing the interaction of observational and interactive learning with cognitive and emotional self-structures. In this model, context is seen as the main driver of development, particularly through observation of others [17].

The second thread of this social-cognitive-ecological framework focuses on the role of the developing self. A key cognitive structure in this model is the child’s developing self-schema and self-identity. Early in an individual’s life, the developing self-schema is influenced by interactions with parents and other primary caregivers. Later, self-schema development is influenced by interactions with peers, schools, and communities, as well as exposure to mainstream cultural beliefs through various media (e.g., books, television, film, etc.; [28]). Huesmann, Dubow, and colleagues’ work focuses primarily on attitudes, beliefs, and behaviors directed at other ethnic groups; thus, it follows that their work tunes in to particular aspects of self-schemas and identity, including ethnic identity values, normative beliefs, and world schemas [17].

Lastly, the third thread of this social-cognitive-ecological model assumes that human development can only be understood by examining how the multiple contexts affecting adolescents and adults in their lives interact to affect individual development. Here, the term “ecological” refers to the nested contexts that constitute an individual’s developmental environment, similar to Bronfenbrenner’s ecological systems theory (1979) [12] outlined above. In this model, it is theorized that no single ecological factor by itself explains more than a small portion of individual differences in behavior or adjustment; instead, multiple factors in concert affect outcomes [17].

## 7. Resilience

Resilience theory provides a conceptual framework for understanding adolescent development from a strengths-based perspective [29,30,31,32,33,34]. Resilience theory supplies the conceptual scaffolding for understanding how and why positive adaptation occurs in spite of exposure to known risk factors [35,36]. This theoretical framework focuses primarily on the contextual, social, and individual variables that disrupt developmental trajectories, from risk to maladaptive outcomes. Some researchers study resilience by examining single risks and promotive factors; however, there has been a shift in focus toward the cumulative effects of several promotive factors across social ecological contexts to better capture the impact of multiple spheres of influence on adolescent development [33].

Additionally, there has been a shift in the field from the historical conceptualization that resilience is an individual character trait toward a view that considers resilience to be comprised of both process and outcome [31,33,37], with recent work reflecting a shift toward a more ecological, process-oriented conceptualization of resilience [33].

Relatedly, Betancourt’s social ecological model of resilience is based on the notion that several protective processes contribute to resilient mental health outcomes when considered through the lens of the individual’s social ecology [38]. They define resilience as the attainment of adaptive social and emotional outcomes despite exposure to considerable risk [38]. They define risk as a psychosocial stressor that disrupts normal functioning [38]. Resilience is composed of both protective factors and protective processes that lead to positive developmental outcomes, and trauma, psychological adjustment, resilience, and the mental health of individuals exposed to risk ought to be viewed as a dynamic process, not a personal trait.

## 8. Intervention and Process-Oriented Research from a Developmental Psychopathology Perspective

Below, we preview new research studies presented in this Special Section from a developmental psychopathology perspective towards identifying the processes associated with the impact of political violence on child adjustment and the translation of research findings into more effective approaches to intervention.

## 9. Intervention Studies

Building on EST and resilience theory, Miller-Graff and Cummings (2022) [39] implemented and evaluated the Promoting Positive Family Futures (PPFF) program, a short family-based coping and support program in Gaza. Sixty-eight families (i.e., mother, father, and one adolescent) were randomized into the PPFF intervention or another longer, locally well-established treatment as usual. Results showed that for both conditions—paternal and maternal depression—emotion regulation using cognitive reappraisal, family-wide emotional security, and adolescent adjustment all improved. Fathers in the PPFF condition reported lower depression and higher emotion regulation at the post-test than did fathers who received treatment as usual. Additionally, mothers in the PPFF condition reported higher levels of emotion regulation at the post-test than did mothers receiving treatment as usual. For both mothers and fathers, PPFF appeared to indirectly improve depression through better emotion regulation using cognitive reappraisal. Lastly, improvements in maternal emotion regulation using cognitive reappraisal were associated with more positive adolescent adjustment at six months. These findings show that the PPFF intervention is beneficial to participants on individual and family levels and is comparable to longer treatment as usual [39].

In a highly innovative approach to intervention, Desrosiers and colleagues (2023) [40] present an exciting intervention strategy for testing the dissemination of evidence-based interventions among youth in low- and middle-income countries to support youth adjustment. The focus was on exploring the diffusion of an evidence-based mental health intervention—the Youth Readiness Intervention (YRI)—among peer networks of Sierra Leonean youth in a trial of a youth employment promotion program, with the YRI delivered with an entrepreneurship training program (N = 165) and including a control program (N = 165). The YRI has been demonstrated to be effective in improving emotion regulation, functional impairment, and pro-social attitudes among high-risk Sierra Leonean youth in secondary schools [14,41,42]. Additionally, the YRI can be delivered by lay workers when supported by robust training and supervision, increasing the range of local individuals who can facilitate the intervention and thus increasing its feasibility [43]. In the present study, participants nominated three of their closest peers, who were then enrolled in the study, with the findings supporting that YRI knowledge was higher among YRI peers compared to peers of controls. Qualitative analyses further supported the diffusion of YRI skills and components across peer networks (e.g., progressive muscle relaxation).

## 10. Process-Oriented Studies

Spiedel and colleagues (2021) assessed social–emotional capacities, including emotion regulation, sympathy, optimism, and trust, as potential moderators between child, parental, and familial pre-migratory adversities and child internalizing and externalizing symptomatology among Syrian refugee children and their mothers living in Canada [44]. Children and mothers reported their pre-migratory adverse life experiences, while mothers reported on their children’s social–emotional capacities, internalizing symptoms, and externalizing symptoms. Results showed that increased familial (i.e., both child and maternal) pre-migratory adversity was linked to more child internalizing and externalizing symptoms post-migration. In contrast, better emotion regulation and optimism were linked to lower internalizing and externalizing symptoms, while higher sympathy was linked to lower levels of externalizing symptomatology. However, higher trust was positively associated with higher internalizing symptoms. Lastly, children’s optimism protected against the association between familial pre-migratory adversity and child internalizing symptoms. This work highlights the protective role certain social–emotional capacities may play in the outcomes of refugee children after resettlement. Additionally, this study illustrates the importance of considering both individual and cumulative, or whole-family, effects of pre-migratory adversity at various levels of the ecological system.

Maloney et al. (2022) [45] explored the links among maternal and paternal trauma exposure, emotional security, and mental health in relation to parent-reported adolescent psychological adjustment while controlling for adolescent trauma exposure and security in the family. The data comes from 68 families (i.e., mother, father, and one adolescent) in the Gaza Strip. Analyses indicate that maternal depression is associated with greater adolescent adjustment and that greater maternal emotional security in the family is related to fewer adolescent adjustment difficulties. Moreover, increased paternal trauma exposure was linked to greater adolescent adjustment problems. This work underscores the importance of understanding family-level factors associated with adolescent adjustment as they relate to addressing the mental health concerns of youth in Gaza [45].

Merrilees et al.’s (2022) [46] manuscript built on a six-wave longitudinal study designed to allow complete tests of relations between political violence and youth adjustment based on EST and a fully articulated social ecological model. They used Time Varying Effects Modeling (TVEM) to advance a novel and rigorous assessment of when, and for whom exposure to ethno-politically motivated (i.e., sectarian) community violence and youth’s mental health problems changed as a function of age across multiple neighborhoods in Belfast, Northern Ireland. Reflecting nonlinear patterns of change across adolescence, the strongest links between exposure to political violence and youth adjustment problems were identified in youth between 16 and 19 years of age. Moreover, ordinary (non-sectarian) community violence was also linked with youth’s mental health problems in later adolescence, but only for Protestant (and not Catholic) youth. These findings thus identify dynamic patterns of change in relations between political violence and youth adjustment, increasing support for the strength of these relations as youth approach adulthood, which has important implications for potential intervention efforts.

Building on a programmatic study of 1051 Israeli and Palestinian youth (ages at Wave 1 ranged from 8 to 14, and at Wave 4 from 15 to 22) over four waves that extensively examines the Social Cognitive Ecological Framework as an explanatory model for relations between political violence and youth adjustment [47,48,49], Huesmann and colleagues (2023) [50] investigated individual dispositions that discriminate between those who act aggressively after exposure to war violence and those more likely to display post-traumatic stress symptoms (PTS). Based on recent theorizing on desensitization and arousal, there is support for the proposition that high anxious arousal can inhibit aggression [49,51,52]. Thus, the hypothesis was that individuals who characteristically experience elevated anxious arousal when exposed to violence will display a greater increase in PTS symptoms and a lower increase in aggression after exposure to war violence. Notably, although exposure to war violence increased both the risk of subsequent aggression and PTS symptoms, anxious arousal in response to a very violent film unrelated to war violence moderated relations with responses to war violence and youth’s reactions, such that those with anxious arousal showed a weaker relationship with aggression and a stronger positive relationship with PTS symptoms.

## 11. Recommendations for the Future

To better understand how exposure to violence in the community affects youth development, more process-oriented longitudinal work that illuminates the mechanisms of action related to both positive and negative outcomes, as well as work that considers the different spheres of youths’ social ecologies, is needed [2,53]. Process-oriented work paves the way for the creation of effective, high-quality interventions geared toward youth affected by political violence; thus, an advanced understanding of the psychological effects on youth at the level of communities and families is crucial to the goal of lasting, sustainable peace in all contexts of political violence. The papers in the current special section highlight some of the ways the field has advanced in doing such process-oriented work on youth impacted by political violence. In terms of future directions, there is a gap in studies that include tests of relations among broader macrolevel factors and youth outcomes (see [54] for an exception). Given the likely widely varying macro-contexts of political violence worldwide, and the likely significance of the macrosystem, an urgent recommendation is for researchers to expand exploration of the role of the macrosystem. In closing, our hope is that this Special Section provides the impetus for the development of new approaches to support more efficacious intervention studies and more informative basic research to provide the foundations for important new advances.

## References

[1] United Nations The Missing Peace: Independent Progress Study on Youth, Peace and Security (2 March 2018). https://www.unfpa.org/sites/default/files/resource-pdf/Progress_Study_on_Youth_Peace_Security_A-72-761_S-2018-86_ENGLISH.pdf.

[2] Cummings E.M., Merrilees C.E., Taylor L.K., Mondi C.F. (2017). Political Violence, Armed Conflict, and Youth Adjustment: A Developmental Psychopathology Perspective on Research and Intervention.

[3] Masten A.S. (2014). Global Perspectives on Resilience in Children and Youth. Child Dev..

[4] Cummings E.M., Merrilees C.E., Taylor L.K., Mondi C.F. (2017). Developmental and social–ecological perspectives on children, political violence, and armed conflict. Dev. Psychopathol..

[5] Barber B.K. (2013). Annual Research Review: The experience of youth with political conflict—Challenging notions of resilience and encouraging research refinement. J. Child Psychol. Psychiatry.

[6] Cummings E.M., Goeke-Morey M.C., Schermerhorn A.C., Merrilees C.E., Cairns E. (2009). Children and Political Violence from a Social Ecological Perspective: Implications from Research on Children and Families in Northern Ireland. Clin. Child Fam. Psychol. Rev..

[7] Betancourt T.S., Borisova I., Williams T.P., Meyers-Ohki S.E., Rubin-Smith J.E., Annan J., Kohrt B.A. (2013). Research Review: Psychosocial adjustment and mental health in former child soldiers—A systematic review of the literature and recommendations for future research. J. Child Psychol. Psychiatry.

[8] Scharpf F., Kaltenbach E., Nickerson A., Hecker T. (2021). A systematic review of socio-ecological factors contributing to risk and protection of the mental health of refugee children and adolescents. Clin. Psychol. Rev..

[9] Tol W.A., Song S., Jordans M. (2013). Annual Research Review: Resilience and mental health in children and adolescents living in areas of armed conflict—A systematic review of findings in low- and middle-income countries. J. Child Psychol. Psychiatry.

[10] Cummings E.M., Davies P.T., Campbell S.B. (2000). Developmental Psychopathology and Family Process: Theory, Research, and Clinical Implications.

[11] Sroufe L.A. (1997). Psychopathology as an outcome of development. Dev. Psychopathol..

[12] Bronfenbrenner U. (1979). Ecology of Human Development: Experiments by Nature and Design.

[13] Bronfenbrenner U. (1986). Ecology of the family as a context for human development: Research perspectives. Dev. Psychol..

[14] Betancourt T.S., McBain R., Newnham E.A., Brennan R.T. (2014). Context matters: Community characteristics and mental health among war-affected youth in Sierra Leone. J. Child Psychol. Psychiatry.

[15] Boxer P., Huesmann L.R., Dubow E.F., Landau S.F., Gvirsman S.D., Shikaki K., Ginges J. (2013). Exposure to Violence Across the Social Ecosystem and the Development of Aggression: A Test of Ecological Theory in the Israeli-Palestinian Conflict. Child Dev..

[16] Cummings E.M., Goeke-Morey M.C., Merrilees C.E., Taylor L.K., Shirlow P.A. (2014). A Social-Ecological, Process-Oriented Perspective on Political Violence and Child Development. Child Dev. Perspect..

[17] Dubow E.F., Huesmann L.R., Boxer P. (2009). A social cognitive-ecological framework for understanding the impact of ex-posure to persistent ethnic–political violence on children’s psychosocial adjustment. Clin. Child Fam. Psychol. Rev..

[18] Cummings E.M., Koss K.J., Davies P.T. (2015). Prospective Relations between Family Conflict and Adolescent Maladjustment: Security in the Family System as a Mediating Process. J. Abnorm. Child Psychol..

[19] Davies P.T., Harold G.T., Goeke-Morey M.C., Cummings E.M., Shelton K., Rasi J.A. (2002). Child emotional security and interparental conflict. Monogr. Soc. Res. Child Dev..

[20] Cummings E.M., Merrilees C.E., Schermerhorn A.C., Goeke-Morey M.C., Shirlow P., Cairns E. (2011). Longitudinal Pathways between Political Violence and Child Adjustment: The Role of Emotional Security about the Community in Northern Ireland. J. Abnorm. Child Psychol..

[21] Cummings E.M., Merrilees C.E., Schermerhorn A.C., Goeke-Morey M.C., Shirlow P., Cairns E. (2012). Political Violence and Child Adjustment: Longitudinal Tests of Sectarian Antisocial Behavior, Family Conflict, and Insecurity as Explanatory Pathways. Child Dev..

[22] Davies P.T., Cummings E.M. (1994). Marital conflict and child adjustment: An emotional security hypothesis. Psychol. Bull..

[23] Cummings E.M., Merrilees C.E., Schermerhorn A.C., Goeke-Morey M.C., Shirlow P., Cairns E. (2010). Testing a social ecological model for relations between political violence and child adjustment in Northern Ireland. Dev. Psychopathol..

[24] Bar-Tal D., Jacobson D. (1998). A psychological perspective on security. Appl. Psychol. Int. Rev..

[25] Batniji R., Rabaia Y., Nguyen-Gillham V., Giacaman R., Sarraj E., Punamaki R.L., Boyce W. (2009). Health as human security in the occupied Palestinian territory. Lancet.

[26] Hobfoll S.E., Hall B.J., Canetti-Nisim D., Galea S., Johnson R.J., Palmieri P.A. (2007). Refining our understanding of traumatic growth in the face of terrorism: Moving from meaning cognitions to doing what is meaningful. Appl. Psychol. Int. Rev..

[27] McAloney K., McCrystal P., Percy A., McCartan C. (2009). Damaged youth: Prevalence of community violence exposure and implications for adolescent well-being in post-conflict Northern Ireland. J. Community Psychol..

[28] Erikson E.H. (1968). Identity: Youth and Crisis.

[29] Fergus S., Zimmerman M.A. (2005). ADOLESCENT RESILIENCE: A Framework for Understanding Healthy Development in the Face of Risk. Annu. Rev. Public Health.

[30] Luthar S.S. (1993). Methodological and Conceptual Issues in Research on Childhood Resilience. J. Child Psychol. Psychiatry.

[31] Luthar S.S., Cicchetti D., Becker B. (2000). The Construct of Resilience: A Critical Evaluation and Guidelines for Future Work. Child Dev..

[32] Luthar S.S., Doernberger C.H., Zigler E. (1993). Resilience is not a unidimensional construct: Insights from a prospective study of inner-city adolescents. Dev. Psychopathol..

[33] Ungar M., Ghazinour M., Richter J. (2013). Annual research review: What is resilience within the social ecology of human development?. J. Child Psychol. Psychiatry.

[34] Zimmerman M.A. (2013). Resiliency Theory. Health Educ. Behav..

[35] Garmezy N., Portney K.E., Berry J.M. (1991). Resiliency and Vulnerability to Adverse Developmental Outcomes Associated with Poverty. Am. Behav. Sci..

[36] Masten A., Cutuli J., Herbers J., Reed J., Lopez S., Snyder C. (2007). Resilience in development. The Oxford Handbook of Positive Psychology.

[37] Miller-Graff L.E. (2022). The Multidimensional Taxonomy of Individual Resilience. Trauma Violence Abus..

[38] Betancourt T., Khan K.T. (2008). The mental health of children affected by armed conflict: Protective processes and pathways to resilience. Int. Rev. Psychiatry.

[39] Miller-Graff L.E., Cummings E.M. (2022). Supporting Youth and Families in Gaza: A Randomized Controlled Trial of a Family-Based Intervention Program. Int. J. Environ. Res. Public Health.

[40] Desrosiers A., Bond L., Hoffman M., Kumar P., Schafer C., Metzger I.W., Vandi A., Hinton M., Betancourt T.S. (2023). Exploring Naturalistic Diffusion of an Evidence-Based Mental Health Intervention across Peer Networks of Youth in Sierra Leone. Int. J. Environ. Res. Public Health.

[41] Betancourt T.S., McBain R., Newnham E.A., Akinsulure-Smith A.M., Brennan R.T., Weisz J.R., Hansen N.B. (2014). A Behavioral Intervention for War-Affected Youth in Sierra Leone: A Randomized Controlled Trial. J. Am. Acad. Child Adolesc. Psychiatry.

[42] Betancourt T., Newnham E., Hann K., Raynaud J., Gau S., Hodes M. (2014). Addressing the consequences of violence and adversity: The development of a group mental health intervention for war-affected youth in Sierra Leone. Research to Practice in Child and Adolescent Mental Health.

[43] Desrosiers A., Freeman J., Mitra R., Bond L., Santo L.D., Farrar J., Borg R., Jambai M., Betancourt T.S. (2022). Alternative Delivery Platforms for Expanding Evidence-based Mental Health Interventions for Youth in Sierra Leone: A Pilot Study. Vulnerable Child. Youth Stud..

[44] Speidel R., Galarneau E., Elsayed D., Mahhouk S., Filippelli J., Colasante T., Malti T. (2021). Refugee Children’s Social–Emotional Capacities: Links to Mental Health upon Resettlement and Buffering Effects on Pre-Migratory Adversity. Int. J. Environ. Res. Public Health.

[45] Maloney C.A., Miller-Graff L.E., Wentz B., Cummings E.M. (2022). Evaluating the Role of Maternal and Paternal Trauma Exposure, Emotional Security, and Mental Health in Predicting Psychological Adjustment among Palestinian Adolescents. Int. J. Environ. Res. Public Health.

[46] Merrilees C.E., Taylor L.K., Goeke-Morey M.C., Shirlow P., Cummings E.M. (2022). Age as a Dynamic Moderator of Relations between Exposure to Political Conflict and Mental Health. Int. J. Environ. Res. Public Health.

[47] Dubow E.F., Huesmann L.R., Boxer P., Landau S., Dvir S., Shikaki K., Ginges J. (2012). Exposure to Political Conflict and Violence and Posttraumatic Stress in Middle East Youth: Protective Factors. J. Clin. Child Adolesc. Psychol..

[48] Dubow E.F., Huesmann L.R., Boxer P., Smith C., Landau S.F., Gvirsman S.D., Shikaki K. (2019). Serious violent behavior and antisocial outcomes as consequences of exposure to ethnic-political conflict and violence among Israeli and Palestinian youth. Aggress. Behav..

[49] Huesmann L.R., Dubow E.F., Boxer P., Landau S.F., Gvirsman S.D., Shikaki K. (2017). Children’s exposure to violent political conflict stimulates aggression at peers by increasing emotional distress, aggressive script rehearsal, and normative beliefs favoring aggression. Dev. Psychopathol..

[50] Huesmann L.R., Dubow E.F., Boxer P., Smith C., Shikaki K., Landau S., Gvirsman S.D. (2023). Consequences of Exposure to War Violence: Discriminating Those with Heightened Risk for Aggression from Those with Heightened Risk for Post-Traumatic Stress Symptoms. Int. J. Environ. Res. Public Health.

[51] Huesmann L.R., Kirwil L., Flannery D.J., Vazsonyi A.T., Waldman I.D. (2007). Why observing violence increases the risk of violent behavior in the observer. The Cambridge Handbook of Violent Behavior and Aggression.

[52] Huesmann L.R., Vazsonyi A.T., Flannery D., DeLisi M. (2018). The contagion of violence. Chapter 30 in A. The Cambridge Handbook of Violent Behavior and Aggression.

[53] Miller-Graff L., Cummings E.M. (2017). The Israeli-Palestinian conflict: Effects on youth, available interventions, and future research directions. Dev. Rev..

[54] Townsend D., Taylor L.K., Merrilees C.E., Furey A., Goeke-Morey M.C., Shirlow P., Cummings E.M. (2020). Youth in Northern Ireland: Linking Violence Exposure, Emotional Insecurity, and the Political Macrosystem. Monogr. Soc. Res. Child Dev..

